# Challenges and Pitfalls in the Management of Rhino-Orbital Mucormycosis in Ophthalmology: A Highlighted Problem in the COVID-19 Era

**DOI:** 10.18502/jovr.v17i3.11582

**Published:** 2022-08-15

**Authors:** Farzad Pakdel, Amin Zand, Ali Sharifi, Masih Asadi, Kaveh Abri Aghdam

**Affiliations:** ^1^Department of Oculo-Facial Plastic Surgery, Department of Ophthalmology, Farabi Hospital, Tehran University of Medical Sciences, Tehran, Iran; ^2^Department of Ophthalmology, Shafa Hospital, Kerman University of Medical Sciences, Kerman, Iran; ^3^Eye Research Center, Eye Department, The Five Senses Health Institute, School of Medicine, Iran University of Medical Sciences, Tehran, Iran

## Abstract

Secondary infections in hospitalized and ill patients with coronavirus disease 2019 (COVID-19) are common. One of these life-threatening infectious diseases is rhino-orbital mucormycosis, which made an outbreak recently. This outbreak was mainly caused by the administration of high-dose corticosteroids in patients with COVID-19, especially those with diabetes mellitus. The increased incidence of rhino-orbital mucormycosis in the COVID-19 era presents different challenges for healthcare providers including ophthalmologists who are directly involved in disease management. We summarized the main challenges and recommendations for ophthalmologists on the management of rhino-orbital mucormycosis.

##  INTRODUCTION

The coronavirus disease 2019 (COVID-19) pandemic has created different challenges for healthcare systems including the occurrence of secondary infections in affected patients. In hospitalized and severely ill patients suffering with COVID-19, secondary infections are more common. Studies have shown that the incidence of fungal infections including candidiasis, aspergillosis, and mucormycosis is cumulatively 10-times more in these patients.^[1,2,3]^ Recently, rhino-orbital mucormycosis impacted a substantial population in several countries. Some studies estimated the pooled prevalence of COVID-19-associated mucormycosis much higher than the highest recorded background of mucormycosis (7/1000 vs 0.14/1000 cases). Most of the cases were diagnosed several days to a few weeks after admission for COVID-19.^[1,2,3][4]^ Mucormycosis is a form of Zygomycosis caused by the Mucorales species of the phylum Zygomycota.^[5]^ It is much more common in immunocompromised humans, especially in patients with diabetes mellitus (DM), leukemia, or lymphoma.^[6]^ The main contributing factors for this deadly disease in the COVID-19 era are the presence of pre-existing immunodeficiency conditions, mainly DM, hematologic, and solid malignancies, the consumption of immunosuppressive and immunomodulatory agents, and the administration of intravenous corticosteroids. Systemic corticosteroids including methylprednisolone and dexamethasone are commonly administered to COVID-19 patients to reduce lung injury and respiratory failure. Following a rapid rise in the incidence of the COVID-19 delta variant in 2021 on an overwhelmed healthcare system, it was observed that a remarkable number of patients received unattended prescriptions of drugs including systemic corticosteroids at home. In addition, unnecessary treatment as well as overtreatment using these agents was seen even in those under professional care.^[1,2,3][4]^ We overviewed different aspects of the challenges and pitfalls in the management of rhino-orbital mucormycosis from the ophthalmology perspective.

### Which Ophthalmic Findings Are Important and Helpful for Making the Diagnosis and Evaluating the Treatment Response in Follow-up Visits?

There are no definite criteria for the diagnosis and management of mucormycosis. Based on reported clinical and paraclinical features of COVID-19-associated mucormycosis and the available guidelines for mucormycosis, the following clinical features should alert the physician for justified intensive interventions:^[2,6,7,8]^

Sudden decrease of vision in any patient with a recent COVID-19 diagnosis;

Recent peri-orbital swelling in any patient with a recent COVID-19 diagnosis;

Orbital or facial pain or headache in any patient with a recent COVID-19 diagnosis;

Blood mixed with nasal discharge;

Drooping of the eyelid, proptosis of the globe, or ophthalmoplegia;

Multiple and unrelated palsies of the cranial nerves;

Black, necrotic tissues in nasal turbinates that are easily mistaken for crusted blood.

In all patients with mucormycosis, whether the orbit is involved or not in the imaging studies, initial and serial comprehensive ophthalmic evaluations include checking the following: visual acuity: relative afferent pupillary defect (RAPD); extraocular motilities; eyelid exam for any subtle changes (e.g., swelling, erythema, or ptosis); globe malpositioning such as proptosis; any ocular surface disorders (e.g., corneal epithelial defects or conjunctival chemosis); eye globe firmness and intraocular pressure (IOP); and a funduscopy exam (for evaluation of optic nerve head, retinal vessels, and retinal status), which are all necessary to be performed by ophthalmologists. We have seen patients with mucormycosis and active proliferative diabetic retinopathy or glaucoma where these sight-threatening ophthalmic situations were missed, because either none or inadequate ophthalmology examinations were done during the disease management.

Other symptoms and signs that must be checked are periorbital, cheek, alveolar, or dental pain which may radiate to the ear, neck, and head. Hypoesthesia/anesthesia over the cheek is a relatively uncommon sign in patients with rhino-orbital mucormycosis and represents the involvement of the infraorbital nerve. Investigation of the hard and soft palate and gingiva mucosa for any necrotic tissue is also helpful for diagnosis. Patients must be visited frequently during the treatment period to assess their response to the treatments and make immediate decisions for further interventions.

### What Are the Imaging Findings of Rhino-Orbital Mucormycosis?

There are no specific signs of rhino-orbital mucormycosis in the computed tomography (CT) scans and magnetic resonance imaging (MRI) of the paranasal sinuses (PNS) and orbit. Mucosal thickening with mild and heterogeneous enhancement in post-contrast PNS CT scans could be seen. Another feature that may be revealed by the imaging is the orbital abscess and its soft tissue thickening. Orbital bony destruction is not common. Isodense lesions relative to muscle/brain and isointense lesions relative to the brain in T1-weighted images are seen on the CT scan and MR images of most patients, respectively, however, the signal intensity in T2-weighted images is variable.^[9]^

The combination of CT scans and MRIs helps the physician to make a more accurate diagnosis and evaluation of the extension of involvement. In patients with signs of intracranial or skull base involvement in imaging (e.g., involvement of pterygopalatine fossa or cavernous sinus), immediate interventions including administration of high doses of an antifungal drug, salvage combination therapy, and surgical debridement should be considered.^[2]^

### Be Aware of Masquerading Diseases!

Abscess formation, tumors (benign or malignant), and other infectious diseases like bacterial or other fungal sinusitis may mimic symptoms and signs of rhino-orbital mucormycosis. Therefore, complete investigation including CT scans and MRIs of the PNS, orbital cavities, and brain, as well as performing a nasal endoscopy are imperative in patients suspicious of having rhino-orbital mucormycosis. Some reports have also shown concomitant mucormycosis with other fungal species including aspergillosis and candidiasis, especially in immunocompromised patients.^[10,11]^ These situations need combined anti-fungal drugs administration to treat the diseases.

### Is Empirical Therapy Recommended?

Although all suspected cases of rhino-orbital mucormycosis need to be confirmed by positive culture and histopathological changes, any delay in the disease management may be associated with irreparable morbidities. On the other hand, medical treatment with antifungal drugs may have serious and even fatal side effects in some patients with certain underlying diseases. In addition, administration of empirical antifungal treatments for suspected patients may have considerable cost to them, especially in low-income countries.^[12,13]^ Therefore, the decision for empirical therapy remains an important challenge for clinicians; multidisciplinary team working for proper diagnosis of disease in suspected cases is also recommended, emphatically. Logically, in suspected cases with disseminated and life-threatening diseases (including cases with signs of intracranial involvement), treatment must be initiated as soon as possible along with the control of other underlying diseases.

### How to Initiate Treatment?

Treatment of rhino-orbital mucormycosis is a combination of surgical debridement of the involved tissues and medical therapy with antifungal drugs. In addition, risk assessment is an important issue in the control of the disease.^[8]^ Consultation with an internist is necessary to control serum glucose levels and diabetic ketoacidosis (DKA). Blood glucose monitoring is a logical step during the treatment.

The drug of choice for initiation of treatment is intravenous amphotericin B. It is a polyene antifungal drug that binds with ergosterol on the fungal cell membranes, forming pores in them and leading to cell death. The drug has two main forms, deoxycholate (conventional) and liposomal. The liposomal form of the drug is superior to the deoxycholate form, because its toxic effects on organs (including the kidney) are fewer, and its efficacy and penetration into the blood–brain barrier and distribution throughout the central nervous system is also more than that of the deoxycholate form.^[14]^

This drug has frequent and significantly adverse effects that need careful monitoring by multiple disciplines including infectious diseases specialists, internists, nephrologists, hematologists, and clinical pharmacologists. Amphotericin B can cause urinary potassium wasting and hypokalemia. So, the drug must be administered slowly with vital signs and cardiac monitoring. Other renal complications of the drug are urinary magnesium wasting and hypomagnesemia, metabolic acidosis, and polyuria due to nephrogenic diabetes insipidus.^[14]^

Due to these adverse effects, serial visits by internists and regular checking of blood pressure, serum potassium (K) and magnesium (Mg), atrial blood gas (ABG), regular renal function laboratory tests including blood urea nitrogen (BUN) and serum creatinine (Cr) are recommended.

The correct dosage preparation of the drug is important in preventing lethal consequences of a drug overdose. The recommended dosage of liposomal amphotericin B for the treatment of rhino-orbital mucormycosis is varied – between 3.0 and 5.0 mg/Kg/day. This dose can be increased to 10 mg/Kg/day for patients with intracranial involvement. The recommended dosage for the deoxycholate (conventional) form is 0.5–1.0 mg/Kg/day.^[14]^ So, the dosage of the deoxycholate form is about one-fifth of the liposomal form. This significant difference is very important and needs to be taken into account during the preparation of the deoxycholate form to prevent the lethal consequences of ingesting an inaccurate dosage. Some reports of cardiac arrest and mortality following amphotericin B overdoses have been reported.^[15]^ Cardiac toxicity is a rare but potentially lethal adverse effect of amphotericin B administration. Ventricular arrhythmias and bradycardia have been reported not only in acute overdose but even with correct dosage with slow rates of drug infusion.^[16,17]^ Previous studies showed the drug itself could be cardiotoxic and these complications were reported even in patients with a normal concentration of potassium and magnesium.^[18]^

The drug could be embryotoxic if administered during pregnancy. The drug is classified as a pregnancy category B by the United States Food and Drug Administration (FDA).^[19]^ An experimental study showed that 0.5 mg/kg of amphotericin B and 2 mg/kg of its methyl derivative can be embryotoxic in rats.^[20]^ So, in pregnant and lactating women with rhino-orbital involvement, treatment initiation with amphotericin B must be done with excessive caution using as low as possible doses. Therefore, planning for the administration of alternative antifungal drugs that have fewer adverse effects must be considered in particular cases.

### When Are Combined Antifungal Treatments Suggested?

Few studies have shown that combination therapy with triazoles and polyenes in patients with disseminated mucormycosis (e.g., intracranial involvement), especially in neutropenic or DKA patients can be beneficial.^[21]^ The suggested drugs to be added to amphotericin B therapy are:

Caspofungin: Combination therapy with caspofungin was associated with significantly improved survival for patients with DKA and rhino-orbito-cerebral mucormycosis.^[22]^ The evidence for use in humans is still weak.

Micafungin: Combination therapy with micafungin improved survival in neutropenic and DKA mice with disseminated mucormycosis.^[23]^ The evidence for use in humans is still weak.

Posaconazole: Combination therapy with oral posaconazole in febrile neutropenic patients with disseminated infection can significantly improve the survival rate.^[24]^

Isavuconazole: It has shown activity against mucormycosis with efficacy similar to amphotericin B. Isavuconazole can be used in combination with amphotericin B for the treatment of rhino-orbital mucormycosis.^[25]^ In addition, posaconazole or isavuconazole can be administrated as salvage therapy for patients who do not respond to or cannot tolerate amphotericin B.^[8]^

### How Long Is the Duration of Treatment?

After the achievement of acceptable clinical responses to the administration of intravenous liposomal amphotericin B, which usually takes more than three to six weeks, patients can be switched to oral posaconazole or isavuconazole as step-down therapy.^[6]^ Treatment must be continued until the resolution of the clinical and radiographic signs of the active disease. It is important to control the underlying causes of the immunosuppression conditions, before treatment cessation.

### When Is Surgical Debridement Necessary?

Necrotic and infected areas in the nasal cavity and PNS should be extensively debrided. Necrotic areas in the orbit and brain may also be debrided limitedly when appropriate. Ophthalmology and otolaryngology consultations are necessary in all suspicious cases of rhino-orbital mucormycosis. Early surgical interventions in PNS (e.g., functional endoscopic sinus surgery: FESS) is one of the essential steps in establishing the diagnosis (by finding necrotic tissues and making specimens for the potassium hydroxide [KOH] wet mount and mycology staining and cultures) and also limiting the disease progression by debridement of necrotic tissues from PNS cavities in early phases. Repetition of PNS mucosa debridement is necessary in progressive and severe cases.^[26]^

### Is Regular Irrigation of the Nasal Cavity and PNS with Amphotericin B Recommended?

Some studies showed that nasal irrigation with 100 or 200 μg/mL of amphotericin B did not create a greater benefit than using normal saline solution after PNS debridement surgery.^[27,28]^ According to the results of an *in vitro* study, irrigation of growth media plates embedded with 10 species of fungi commonly found in the nasal cavity, with 20 mL of amphotericin B at concentrations of 200 or 300 μg/mL (twice per day for six weeks), prevented fungal growth successfully.^[29]^ Therefore, the possible benefits of this route of amphotericin B administration are limited and adjuvant regular irrigation of the nasal cavity and PNS with the drug is controversial.

### When Are Topical Medications for the Ocular Surface Problems Necessary?

In patients with orbital mucormycosis, ocular surface complications including dry eye, corneal epithelial erosions and defects, and conjunctival chemosis must be managed carefully with topical lubricants, emollients, and antibiotics.

### When Is the Retrobulbar Injection of Amphotericin B Indicated?

Retrobulbar injection of amphotericin B may facilitate improvement of orbital mucormycosis, in combination with systemic antifungal drugs, and reduce the rate of aggressive interventions including orbital tissues debridement or exenteration. Retrobulbar injections of amphotericin B are recommended in patients with signs of optic nerve compression including progressive decrease in visual acuity and positive RAPD. In addition, this route of drug administration is recommended when other symptoms and signs of orbital involvement including pain, proptosis, globe firmness, limitation of extraocular motility or conjunctival chemosis, and ocular surface exposure have progressed despite complete systemic medical and surgical interventions.^[30]^ This route of drug delivery is reported to be effective in the improvement of cerebritis in patients with intracranial involvement.^[31]^ Retrobulbar injection of the drug is a suitable treatment option for cases in which orbital surgical intervention is not possible, especially for patients admitted to an intensive care unit (ICU). In the COVID-19 era, a high portion of cases were ICU-admitted and were not candidates for surgical intervention (including FESS and orbital surgery). Therefore, this route of administration is a good and superior choice for that group of patients. Reports showed that well-timed decisions for this type of drug administration is important to achieve acceptable results. The recommended dosage for the retrobulbar injection is 3.3–3.5 mg/1 ml of liposomal (preferred) amphotericin B. There is no protocol for the frequency of the drug injections, but some reports recommended injections at least three times during the course of three days, continuously.^[32]^ However, this route of drug administration has some limitations. First, retrobulbar injections are a risky intervention that may cause globe perforation by the syringe needle, retrobulbar hemorrhage, and even inadvertent drug administration to the brain stem. Second, amphotericin B is neurotoxic, and this toxicity is much higher in the conventional form of the drug as compared to the liposomal form.^[32,33]^ Therefore, even with the administration of a correct dosage of liposomal amphotericin B, the possibility of the occurrence of toxic optic neuropathy should be considered. Third, in some reports, the retrobulbar injection of the drug caused orbital compartment syndrome.^[34]^ Fourth, local injection of amphotericin B (especially the deoxycholate form) may induce inflammation by causing soft-tissues edema, especially after the first injection.^[14]^ However, this side effect is usually transient and self-limited. Fifth, amphotericin B has a high molecular weight and drug distribution in orbital connective and fatty tissues is slow and limited.^[32]^ As a consequence, multiple injections are necessary and logical. The previously mentioned adverse effects limit the frequencies of the drug administration to the orbital cavity via the retrobulbar route. The senior author (FP) has gained experience with the administration of a higher frequency of injections at different levels of dosages. It should be mentioned that the retrobulbar injection of amphotericin B is rapidly becoming a standard practice.

### What Is the role of Conservative Orbital Debridement and Adjuvant Local Amphotericin B Irrigation?

In a retrospective study by Seiff et al, on seven immunocompromised patients with rhino-orbital mucormycosis and good visual acuity, surgical debridement of necrotic tissue of the involved PNS and orbits were done. Then, a catheter was inserted and placed into the orbital cavities for regular irrigation of them with amphotericin B solution (with volume of 3–4 ml, and concentration of 0.25–1.00 mg/ml, four times per day, for 5–14 days). They claimed this technique improved the therapeutic outcomes. Joos et al reported successful management of a patient with rhino-orbital mucormycosis with the similar technique (surgical debridement of necrotic tissues, and then regular irrigation of the involved orbital cavity with 5 ml [1 mg/ml] non-liposomal amphotericin B, once a day for seven days).^[35,36]^ Nowadays, this technique is not popular among oculoplastic surgeons, because the benefits of this technique in comparison to its potential risks are low. Some of these potential risks are including uneventful and iatrogenic harms to the orbital components (e.g., nerves and extraocular muscles), creating a route for the entrance of other pathogens, and possible ocular toxicity by the drug. Moreover, the sufficiency of the whole necrotic tissue debridement process and the adequate distribution of the drug into the orbital cavity is under question.

### When Is Orbital Exenteration Indicated?

One of the most challenging decisions in the management of orbital mucormycosis is planning for exenteration in those cases where the nature of the disease is life-threatening. Here, the main questions are whether exenteration can reduce the mortality rate and whether the benefits of exenteration are satisfactory enough against its lifetime disabling effects. There is no standard protocol for choosing to perform orbital exenteration in patients with rhino-orbital mucormycosis; it is mainly dependent on the judgment of the physician. The most important deciding factors for performing exenteration in patients with orbital mucormycosis are the presence of ophthalmoplegia and cranial nerves palsy, proptosis, ocular tissues involvement, progressive visual impairment or vision loss, general necrosis of the orbital cavity and its adnexa, major immunosuppressive conditions including underlying diseases and neutropenia, cerebral extension, prolonged disease despite adequate medical and surgical interventions, and unsatisfactory response to the initial treatments.^[37,38]^

Generally, in cases resistant to all medical and surgical interventions with progressive signs and symptoms of the disease, especially the probability of intracranial involvement and fulminant infection, some physicians recommended orbital exenteration to decrease the mortality risk. However, a review on indications of orbital exenteration in patients with mucormycosis revealed there are no standard guidelines for making decision of exenteration in these patients. They showed that just exenterated patients with fever were more likely to survive compared with non-exenterated febrile cases.^[38]^

### Which Care Measures Are Necessary After Orbital Exenteration?

After orbital exenteration, patients need frequent socket examinations for the occurrence of any infections including the recurrence of the fungal infection. The socket should be inspected and cleaned with diluted betadine solution for the first three weeks until the surface is adequately epithelialized. In some patients with superficial recurrence of mucormycosis, regular irrigation with an amphotericin B solution is recommended. Sometimes, debridement of remnant necrotic tissues of the orbital cavity is necessary.^[39]^

Many patients will be dissatisfied with the cosmetic outcomes of the surgery. This condition creates significant psycho-social challenges for the patients. Therefore, a counseling plan for the patients is necessary to rehabilitate their cosmesis. Prosthetic reconstruction of orbital defects with facial plastic surgeries is suggested.^[40]^

### Which Variables Are More Important in Patients' Survival?

In a review by Hargrove et al,^[38]^ the investigators revealed that patients aged > 46 years with frontal sinus involvement and fever had less survival rates in comparison to those without these conditions, regardless of performing orbital exenteration in patients with rhino-orbital mucormycosis. In addition, they showed patients treated with amphotericin B and those with diabetes were more likely to survive compared with those without these conditions.

### What Has Been the Impact of the COVID-19 Pandemic on the Prevalence of Mucormycosis? 

According to a meta-analysis by Hussain et al,^[41]^ the pooled prevalence of rhino-orbito-cerebral mucormycosis among hospitalized COVID-19 patients was 7 cases per 1000 patients. This rate was much higher in comparison to the prevalence of the disease among comorbid populations before the COVID-19 pandemic (0.14/1000).^[42,43]^ In addition, the estimated global incidence rate of mucormycosis varies between 0.005 and 1.7 per million population.^[44]^ Therefore, a very significant surge in the disease's prevalence occurred during the COVID-19 era, and lead to an additional burnout and workload among healthcare providers. In conclusion, management of patients with rhino-orbital mucormycosis is challenging. The first step is making the accurate diagnosis of the disease, and differentiate it from other possible debilitating ocular comorbidities. After making the diagnosis, well-timed management of the disease is life-saving, and needs a multidisciplinary teamwork including infectious diseases specialists, otolaryngologists, ophthalmologists, and internists. Due to unpredictable and mysterious progression of the disease, patients should receive regular follow-up visits to assess and timely revise the treatment plan. By considering the important adverse effects of amphotericin B on serum electrolytes, heart, and kidney, regular monitoring (by monitoring the vital signs, laboratory testing, and electrocardiography) is necessary for these patients. The current management protocols of the disease needs review to make them safer with more cost-effectiveness outcomes (e.g., focusing on combined systemic antifungal regimens and locally targeted administration of the drug in addition to systemic medications). Also, introducing guidelines for controlling the load of intravenous steroids (including dexamethasone, or methylprednisolone) administration in COVID-19 patients seems to be necessary.

##  Financial Support and Sponsorship

None.

##  Conflicts of Interest 

None.
